# The relationships of character strengths with coping, work-related stress, and job satisfaction

**DOI:** 10.3389/fpsyg.2015.00165

**Published:** 2015-02-26

**Authors:** Claudia Harzer, Willibald Ruch

**Affiliations:** ^1^Psychological Assessment, Department of Psychology, University of KasselKassel, Germany; ^2^Personality and Assessment, Department of Psychology, University of ZürichZürich, Switzerland

**Keywords:** character strengths, coping, stress, job satisfaction, nurses, positive psychology

## Abstract

Personality traits have often been highlighted to relate to how people cope with stressful events. The present paper focuses on character strengths as positive personality traits and examines two basic assumptions that were derived from a core characteristic of character strengths (i.e., to determine how individuals deal with adversities): (1) character strengths correlate with coping and (2) buffer the effects of work-related stress on job satisfaction. Two different samples (i.e., a mixed sample representing various occupations [*N* = 214] and a nurses sample [*N* = 175]) filled in measures for character strengths, coping, work-related stress, and job satisfaction. As expected, intellectual, emotional, and interpersonal strengths were related to coping. Interpersonal strengths played a greater role for coping among nurses, as interactions with others are an essential part of their workday. Furthermore, intellectual strengths partially mediated the negative effect of work-related stress on job satisfaction. These findings open a new field for research on the role of personality in coping with work-related stress. Character strengths are trainable personal characteristics, and therefore valuable resources to improve coping with work-related stress and to decrease the negative effects of stress. Further research is needed to investigate this assumed causality.

## INTRODUCTION

Within the work-context, work-related stress is an issue with a strong impact on employees, organizations, and the communities (e.g., Vagg and Spielberger, 1998; Hodapp et al., 2005). Stress occurs when a person “is hard-pressed to deal with some obstacle or impediment or looming threat” (Carver and Connor-Smith, 2010, p. 684). Typical work-related stressors are, for example, workload, time pressure, and conflicts with co-workers (Vagg and Spielberger, 1998). Work-related stress often results in employee dissatisfaction, lowered productivity, absenteeism, and turnover (e.g., Landsbergis, 1988; Karasek and Theorell, 1990; Cooper and Cartwright, 1994). People cope with stress in different ways to prevent or diminish it directly (i.e., reduce the stressor) or indirectly (i.e., reduce associated distress; Carver and Connor-Smith, 2010). Personality traits have often been highlighted to relate to how people cope with stressful events (e.g., Grant and Langan-Fox, 2006; Connor-Smith and Flachsbart, 2007).

The present paper focuses on character strengths as positive personality traits. One of the core defining characteristics of character strengths is that they determine “how an individual copes with adversity” (Peterson and Seligman, 2004, p. 17). Hence, character strengths should (1) be directly related to coping behavior and (2) protect against the negative effects of work-related stress on job satisfaction like coping does (e.g., Kirkcaldy et al., 1995; Wolfgang, 1995). The present paper is aimed at examining whether empirical data support these two assumptions by studying the relationships between character strengths, coping behavior, work-related stress, and job satisfaction. If this is the case, then character strengths, as trainable personal characteristics (Peterson and Seligman, 2004), might function as important resources for the training on and/or off the job in the future. Such training could improve coping with work-related stress in order to decrease the negative consequences of work-related stress for all – the employee, the organization, and the community. Moreover, we aimed at utilizing two samples. One of the samples should be comprised of employees from various occupations in order to study the relationships between the variables of interest on a more general level. The second sample should be a sample of nurses, because this is one of the occupational groups especially exposed to work-related stress, and where coping with stress plays an important role (e.g., Landsbergis, 1988; Greenglass and Burke, 2000). Utilizing these two samples would help to identify replicable relationships between character strengths and coping, but also to have a first insight in job-group specific associations.

### CHARACTER STRENGTHS

Character strengths are positively valued, narrow personality characteristics (e.g., being friendly, honest, and/or persistent, appreciating excellent performances). According to Peterson and Seligman (2004), character strengths are trait-like and valued in their own right. They are not engaged in for the tangible outcomes they may produce, although character strengths do produce desirable outcomes. Character strengths manifest in individual behaviors (e.g., working well in a team), thoughts (e.g., looking positively ahead), and feelings (e.g., being grateful for getting a scholarship). They are seen as the inner determinant of a satisfied, happy, and successful life (i.e., the good life), in addition to external factors like a good education, stable social environment, or financial security (cf. Peterson, 2006). Character strengths are considered to be the components of a positive, good character. For a comprehensive description of a positive, good character, Peterson and Seligman (2004) developed a catalog of 24 different character strengths – the Values in Action (VIA) classification (see **Table 1** for an overview and the definitions of the character strengths).

**Table 1:** The 24 character strengths included in the Values in Action classification of strengths (Peterson and Seligman, 2004) and short descriptions defining the strengths.

**(1) Strengths of wisdom and knowledge (i.e., cognitive strengths that entail the acquisition and use of knowledge)**
*•Creativity [originality, ingenuity]*: Thinking of novel and productive ways to conceptualize and do things; includes artistic achievement but is not limited to it
*•Curiosity [interest, novelty-seeking, openness to experience]*: Taking an interest in all of on-going experience for its own sake; finding subjects and topics fascinating; exploring and discovering
*•Judgment & Open-Mindedness [critical thinking]*: Thinking things through and examining them from all sides; not jumping to conclusions; being able to change one’s mind in light of evidence; weighing all evidence fairly
*•Love of Learning*: Mastering new skills, topics, and bodies of knowledge, whether on one’s own or formally; obviously related to the strength of curiosity but goes beyond it to describe the tendency to add systematically to what one knows
*•Perspective [wisdom]*: Being able to provide wise counsel to others; having ways of looking at the world that make sense to oneself and to other people
**(2) Strengths of courage (i.e., emotional strengths that involve the exercise of will to accomplish goals in the face of opposition, external or internal)**
*•Bravery [valor]*: *Not* shrinking from threat, challenge, difficulty, or pain; speaking up for what is right even if there is opposition; acting on convictions even if unpopular; includes physical bravery but is not limited to it
*•Perseverance [persistence, industriousness]*: Finishing what one starts; persisting in a course of action in spite of obstacles; “getting it out the door”; taking pleasure in completing tasks
*•Honesty [authenticity, integrity]*: Speaking the truth but more broadly and presenting oneself in a genuine way and acting in a sincere way; being without pretense; taking responsibility for one’s feelings and actions
*•Zest [vitality, enthusiasm, vigor, energy]*: Approaching life with excitement and energy; not doing things halfway or half-heartedly; living life as an adventure; feeling alive and activated
**(3) Strengths of humanity (i.e., interpersonal strengths that involve “tending and befriending” others)**
*•Capacity to Love and Be Loved [short name: love]*: Valuing close relations with others, in particular those in which sharing and caring are reciprocated; being close to people
*•Kindness [generosity, nurturance, care, compassion, altruistic love, “niceness”]*: Doing favors and good deeds for others; helping them; taking care of them
*•Social Intelligence [emotional intelligence, personal intelligence]*: Being aware of the motives and feelings of other people and oneself; knowing what to do to fit into different social situations; knowing what makes other people tick
**(4) Strengths of justice (i.e., civic strengths that underlie healthy community life)**
*•Teamwork [citizenship, social responsibility, loyalty]*: Working well as a member of a group or team; being loyal to the group; doing one’s share
*•Fairness:* Treating all people the same according to notions of fairness and justice; not letting personal feelings bias decisions about others; giving everyone a fair chance
*•Leadership*: Encouraging a group of which one is a member to get things done and at the same time maintain good relations within the group; organizing group activities and seeing that they happen
**(5) Strengths of temperance (i.e., strengths that protect against excess)**
*•Forgiveness & Mercy*: Forgiving those who have done wrong; accepting the shortcomings of others; giving people a second chance; not being vengeful
*•Modesty & Humility*: Letting one’s accomplishments speak for themselves; not regarding oneself as more special than one is
*•Prudence*: Being careful about one’s choices; not taking undue risks; *not* saying or doing things that might later be regretted
*•Self-Regulation [self-control]*: Regulating what one feels and does; being disciplined; controlling one’s appetites and emotions
**(6) Strengths of transcendence (i.e., strengths that forge connections to the larger universe and provide meaning; theological strengths)**
*••Appreciation of Beauty and Excellence [awe, wonder, elevation; short name: beauty]*: Noticing and appreciating beauty, excellence, and/or skilled performance in various domains of life, from nature to art to mathematics to science to everyday experience
*•Gratitude*: Being aware of and thankful for the good things that happen; taking time to express thanks
*•Hope [optimism, future-mindedness, future orientation]*: Expecting the best in the future and working to achieve it; believing that a good future is something that can be brought about
*•Humor [playfulness]*: Liking to laugh and tease; bringing smiles to other people; seeing the light side; making (not necessarily telling) jokes
*•Religiousness & Spirituality [faith, purpose]*: Having coherent beliefs about the higher purpose and meaning of the universe; knowing where one fits within the larger scheme; having beliefs about the meaning of life that shape conduct and provide comfort

*The character strengths are grouped together content-wise on a theoretical basis.*

Research showed that the character strengths presented in **Table 1** do contribute to a good, satisfied, and successful life on and off the work-context. For example, the character strengths zest, hope, gratitude, curiosity, love, religiousness, and humor were the ones most robustly related to job satisfaction across job categories (e.g., professional, blue collar, and homemaker; Peterson et al., 2010). Furthermore, different character strengths were meaningfully associated with different work-related behavior. For example, perseverance, zest, and love of learning showed the numerically strongest relationships with career ambition, and employees with higher scores in the character strengths (e.g., hope, zest, bravery, and perspective) tended to have healthier work behavior (Gander et al., 2012). Harzer and Ruch (2014) reported various, replicable associations between character strengths and self- and supervisory ratings of different dimensions of job performance (i.e., task performance, job dedication, interpersonal facilitation, as well as organizational support). For example, task performance was related to perseverance, teamwork, honesty, prudence, and self-regulation. Interpersonal facilitation correlated with teamwork, kindness, leadership, and fairness.

The current “gold standard” of the subjective assessment of the 24 character strengths in adults is the Values in Action Inventory of Strengths (VIA-IS; Peterson et al., 2005). Independent from the original classification of character strengths (cf. **Table 1**), which was done theoretically, on a content-related basis, analyses of the factor structure of the character strengths in adults measured with the VIA-IS were computed (e.g., Peterson, 2006; Peterson et al., 2008; Brdar and Kashdan, 2010). Results differed with respect to the characteristics of (a) samples (e.g., adult volunteers vs. students), (b) data (i.e., absolute vs. ipsative [intra-individually standardized] scores), (c) version of the VIA-IS (e.g., original vs. items from the International Personality Item Pool by Goldberg), and (d) language (e.g., participants filled in the VIA-IS in their native language vs. foreign language; cf. Harzer, 2012). When examining absolute scores (utilizing principal component analysis with Varimax rotation) in non-student samples that filled in the original VIA-IS in a version of their native language, a five factor solution seemed to be the most appropriate one (no matter if data comes from self- or peer-ratings; e.g., Peterson et al., 2008; Ruch et al., 2010; Harzer and Ruch, 2014). These five factors could also be replicated across various German-speaking samples (e.g., Ruch et al., 2010; Proyer and Ruch, 2011; Güsewell and Ruch, 2012; Harzer and Ruch, 2014). The five factors were labeled as *emotional strengths* (also named strengths of fortitude; e.g., loaded by the character strengths bravery, zest, hope, honesty, perspective), *interpersonal strengths* (e.g., capacity to love and be loved, kindness, leadership, teamwork, humor; mainly a combination of interpersonal and civic strengths), *strengths of restraint* (also labeled as temperance; e.g., prudence, forgiveness, fairness, modesty), *intellectual strengths* (also named cognitive strengths; e.g., creativity, curiosity, love of learning), *and theological strengths* (also labeled as transcendence; i.e., appreciation of beauty and excellence, gratitude, religiousness). The present paper focuses on these five factors rather than on 24 character strengths, in order to get a general overview on the relationships between character strengths and coping with stress.

Previous publications on cross-sectional data showed that character strengths are associated with dealing positively with trauma (Peterson and Seligman, 2003; Peterson et al., 2008) and with recovery from illness (Peterson et al., 2006). Especially, intellectual, emotional, and interpersonal strengths were related to dealing with adversity (i.e., trauma and illness). For example, intellectual and interpersonal strengths increased with the number of traumatic events experienced (e.g., life-threatening accident, sexual assault, and physical assault; Peterson et al., 2008). Furthermore, intellectual and emotional strengths tended to be more pronounced in those who had recovered from physical illness compared to those who did not recover (fully) or have not had an illness (Peterson et al., 2006).

The research presented so far shows that character strengths are associated with dealing positively with adversity. However, the relationships between character strengths and coping behavior have never been examined directly. The present paper, therefore, is aimed at examining the relationships between character strengths and coping behaviors to further investigate the role of character strengths in dealing with stress.

### COPING WITH STRESS

This paper focuses on dispositional coping, which is defined as an individual’s habitual way of reacting to stressors with certain coping mechanisms or strategies (i.e., the individual’s characteristic reaction to stressful events; Janke et al., 1985; Janke and Erdmann, 2008). Janke and colleagues provided an extensive model of dispositional coping (e.g., Janke et al., 1985; Janke and Erdmann, 2008). They distinguish between 20 different coping modes, which in turn can be subsumed into two broad categories, namely positive and negative coping strategies (plus a group of four equivocal coping modes, which are not of interest here). *Negative coping strategies* (NEG) entail coping behaviors that do not reduce stress/strain in the long run but augment it (i.e., escape, social withdrawal, rumination, resignation, self-pity, self-blame). *Positive coping strategies* (POS) are assumed to reduce stress; they can be further separated into three subcategories. The first subcategory, called *devaluation/defense* (POS1), covers a cognitive way of coping and entails the coping modes minimization (of intensity, duration, or importance of stress), self-aggrandizement by comparison with others (i.e., attribute less stress to oneself than to others), and denial of guilt. The second one, *distraction* (POS2), is characterized by seeking distraction from strain by focusing on situations and states that are incompatible with stress. It entails the four coping modes distraction (i.e., focus the attention on something else), substitute gratification (i.e., turn to something positive), search for self-affirmation, and relaxation. *Control* (POS3) represents the third positive coping subcategory and entails the active control of stressors and reactions. The related coping modes are situation control (i.e., analyze, plan, and act for control and problem solving), reaction control (of own responses), and positive self-instructions (i.e., to accredit oneself competence and the ability to control).

Research that underlined the validity of the distinction between positive coping as being adaptive, and negative coping as being maladaptive was mainly conducted in the clinical setting (e.g., Grüsser et al., 2006; Möller-Leimkühler, 2006). Research conducted in the work-context showed that higher scores in negative coping predicted lower novices’ performance in surgery (Hassan et al., 2006; Maschuw et al., 2011).

### CHARACTER STRENGTHS AND COPING WITH STRESS

Given the results of previous research on the relationships of character strengths with recovery from illness and trauma, it was expected that especially intellectual, emotional, and interpersonal strengths relate to dispositional coping behavior. There should be a direct relation with positive coping strategies (i.e., positive correlations) and an inverse relation for negative coping strategies (i.e., negative correlations). This was expected, because it was postulated that character strengths contribute to individual fulfillment (cf. Peterson and Seligman, 2004; Peterson, 2006). Therefore, character strengths should be positively related to positive, stress-reducing coping, and negatively to negative, not stress-reducing coping.

More specific hypotheses on the relationships between character strengths and coping were formulated content-driven. *Intellectual strengths* foster the production of new and reasonable strategies for problem solving and the exploration of situational circumstances (e.g., being curious and thinking creatively). This analytical behavior should assist in the learning process regarding what will help to reduce stress and what will not. The following hypothesis was therefore proposed:

Hypothesis 1: Intellectual strengths correlate positively with every subcategory of stress-reducing coping (i.e., [a] POS1, [b] POS2, and [c] POS3).

*Emotional strengths* include active behaviors (e.g., being brave, persistent, and hopeful, having perspective), which should be beneficial for behaviors associated with control (POS3; i.e., analyzing the situation, problem solving, controlling own reactions, and facing a stressful event), rather than engaging in more passive devaluation/defense (POS 1) and distraction (POS 2). Emotional strengths are therefore expected to show stronger relations to the positive coping strategy control (POS3) compared to the strategies devaluation/defense (POS1) and distraction (POS2).

Hypothesis 2: Emotional strengths correlate more strongly with control (POS3) than with (a) devaluation/defense (POS1) and (b) distraction (POS 2).

*Interpersonal strengths* might be especially helpful in dealing positively with stressors in social interactions. Interpersonal strengths might, therefore, play a special role in jobs with a high rate of social interactions (e.g., teachers, nurses, sales persons). Nurses are one of those occupational groups working in a job that is known to be very stressful and where coping with stress plays an important role (e.g., Landsbergis, 1988; Greenglass and Burke, 2000). An often-observed stressor for nurses is the (sometimes) problematic contact with doctors as well as patients and their relatives (e.g., Harris, 1989; Burgess et al., 2010). Hence, interpersonal strengths are expected to be stronger related to coping in nurses than in a mixed sample of employees because interpersonal strengths help to deal with interpersonal conflicts or might even prevent them. The role of interactions with others levels out in mixed samples, and hence the role of interpersonal strengths levels out.

Hypothesis 3: Interpersonal strengths correlate more strongly with positive coping (POS) in a sample of nurses than in a mixed sample.

### CHARACTER STRENGTHS, STRESS, AND JOB SATISFACTION

If character strengths are indeed related to coping, they should also buffer the negative effects of work-related stress on job satisfaction. Studies showed that character strengths are positively related to job satisfaction (e.g., Peterson et al., 2010; Gander et al., 2012). So far, it is not known how character strengths relate to stress at work. However, it has been shown that character strengths buffer the negative effects of an illness on life satisfaction (Peterson et al., 2006). These findings indicated that character strengths might increase with the challenges experienced (e.g., illness, trauma), which in turn are positively related to life satisfaction. Based on those results, it is expected that character strengths might buffer the negative effect of work-related stress on job satisfaction (cf. Landsbergis, 1988; Wolfgang, 1995). It can be expected, that character strengths might profit from the challenges provided by work-related stress. Facing challenges could be seen as a natural learning environment to enhance character strengths, because behavior related to the character strengths might be beneficial in solving the challenges successfully. Therefore, a positive relationship between character strengths and frequency of work-related stress was expected. Fostering character strengths should in turn enhance job satisfaction, and therefore, a (partial) mediation of the negative relationship between frequency of work-related stress and job satisfaction by character strengths was expected.

Nevertheless, a moderation effect might occur as well; that is, character strengths might influence the relation between work-related stress and job satisfaction. For people with high scores in character strengths, work-related stress might have a smaller impact on job satisfaction than for people low in character strengths. Therefore, the mediation and the moderation effect of character strengths on the relationship between work-related stress and job satisfaction was examined. The following, explorative hypotheses were formulated:

Hypothesis 4 (explorative): Character strengths mediate the relationship between the frequency of work-related stress and job satisfaction.

Hypothesis 5 (explorative): Character strengths moderate the relationship between the frequency of work-related stress and job satisfaction.

### AIMS OF THE PRESENT STUDY

The present study had two main aims. Firstly, this study was aimed at investigating the relationships between character strengths and coping in a mixed sample with employees from different occupations and in a sample with nurses. Utilizing the two samples would help to identify replicable relationships between character strengths and coping, but also to have a first insight in job-group specific associations. Analyses will be done on the level of character strengths factors (i.e., emotional strengths, interpersonal strengths, strengths of restraint, intellectual strengths, and theological strengths) and coping strategies (i.e., negative coping and positive coping as well as the three subcategories of positive coping: devaluation/defense, distraction, and control). Secondly, it will be examined whether character strengths mediate and/or moderate the relationship between work-related stress and job satisfaction.

## MATERIALS AND METHODS

### PARTICIPANTS

#### Sample 1 (mixed sample)

The sample consisted of 214 German-speaking adult volunteers (71 men, 143 women). Their mean age was 38.28 years (*SD* = 10.51; range: 21–64 years). Most of the participants were married (*n* = 90) or in a relationship (*n* = 55), *n* = 53 were single, *n* = 12 were separated or divorced, and *n* = 4 were widowed. Participants were highly educated, as *n* = 120 indicated having a Master’s degree, *n* = 37 a doctor’s degree; *n* = 32 had an apprenticeship, *n* = 12 had a school diploma, and *n* = 13 had completed secondary school. Participants represented a wide array of occupations (e.g., office workers, teachers, and researchers). Participants at least worked 50% of full time hours with two third of them working 100% (full-time; *M*_percentage of employment_ = 88.33%, *SD* = 18.73).

#### Sample 2 (nurses sample)

The sample consisted of 175 German-speaking hospital nurses (11 men, 164 women; representing the typical gender ratio in this occupation) from different hospitals. Their mean age was 40.16 years (*SD* = 10.06; range: 21–61 years). Most of the participants were married (*n* = 76) or in a relationship (*n* = 47), and *n* = 33 were single, *n* = 17 were separated or divorced, and *n* = 2 were widowed. Concerning educational level, *n* = 123 had an apprenticeship, *n* = 32 had a Master’s degree, *n* = 13 had completed the secondary school, *n* = 6 had a school diploma allowing them to attend university, and *n* = 1 had a doctor’s degree. Participants at least worked 50% of full time hours with two third of them working 80% (*M*_percentage of employment_ = 83.19%, *SD* = 16.04; range: 50–100%).

The two samples did not differ with respect to age (*t*[387] = –1.79, *p* = 0.074) and marital status (χ^2^[4] = 4.12, *p* = 0.390) but in gender ratio (χ^2^[1] = 41.85, *p* < 0.001), education (χ^2^[5] = 138.34, *p* < 0.001), and percentage of employment (*t*[386.16] = 2.92, *p* = 0.004). Sample 1 (mixed sample) entailed more males, was better educated, and had a higher percentage of employment than sample 2 (nurses sample). Therefore, gender, education, and percentage of employment were controlled in all subsequent analyses.

### INSTRUMENTS

The *Values in Action Inventory of Strengths* (*VIA-IS;* Peterson et al., 2005) is a questionnaire consisting of 240 items in a 5-point Likert-scale answer format (from 1 = *very much unlike me* through 5 = *very much like me*) measuring the 24 character strengths of the VIA classification (10 items per strength, responses are averaged to compute the scale scores). A sample items is “I never quit a task before it is done” (perseverance). The VIA-IS has widely been used in research (e.g., Brdar and Kashdan, 2010; Harzer and Ruch, 2012; Littman-Ovadia and Lavy, 2012; Proyer et al., 2013a). The German version of the VIA-IS (Ruch et al., 2010) showed high internal consistencies (median α = 0.77) and high stability over 9 months (median test–retest correlation = 0.73). Self- and peer-rating forms correlated in the expected range (median correlation = 0.40). In the present study, internal consistencies had a median of 0.78 and 0.74 in sample 1 (mixed sample) and 2 (nurses sample), respectively. The 24 VIA-IS scales were reduced to five strengths factors (i.e., emotional strengths, interpersonal strengths, strengths of restraint, intellectual strengths, and theological strengths) by principal component analysis, subsequent Varimax rotation, and saving the factor scores for further analyses. The factor analysis resulted in five factors that were highly similar to the solution reported by Ruch et al. (2010). The Tucker’s phi coefficients for the corresponding factors ranged from 0.91 to 0.99.

The *Stress Coping Inventory* (*SVF120;* Janke and Erdmann, 2008) is a questionnaire in German language consisting of 120 items in a 5-point Likert-scale answer format (from 0 = *not at all* through 4 = *very likely*) measuring dispositional coping. Sample items are “I plan how to solve the difficulties involved” (situation control). Scores can be computed for 20 coping strategies (i.e., modes measured with six items each, responses are summed up to compute the scale scores), which can be subsumed to two broad categories (i.e., positive strategies [average score of 10 modes] and negative strategies [average score of 6 modes]) and a group of equivocal modes (4 modes). The positive strategies can be separated into the subcategories devaluation/defense, distraction, and control (i.e., average score of three to four modes each). The SVF120 (modes and [subcategories of] strategies) showed to be reliable (median α = 0.84), stable (median test–retest correlation = 0.77), and construct valid (e.g., factorial structure; convergent and discriminant validity; Trempa et al., 2002; Janke and Erdmann, 2008). The SVF120 has been used widely in research (e.g., Möller-Leimkühler, 2006; Maschuw et al., 2011). In the present study, internal consistencies of the 20 coping modes had a median of 0.82 in each of the two samples.

The *Job Stress Survey* (*JSS;* Spielberger and Vagg, 1999) is a questionnaire assessing the frequency (1 = *never* to 9 = *all the time* experienced during the last 6 months) and perceived severity (1 = *least stressful* to 9 = *most stressful*) of 30 job-related events that are stressful for employees in a variety of occupations. Sample stressors are “meeting deadlines,” “excessive paperwork,” and “poorly motivated co-workers.” The German version of the JSS showed high reliability (α ≥ 0.92) and factorial validity (Hodapp et al., 2005). The JSS has widely been used in research (e.g., De Fruyt and Denollet, 2002; Bongard and al’Absi, 2005; Lau et al., 2006). In the present study, the JSS frequency scale was of interest (computed by averaging the frequency ratings of the 30 stressful job-related events). Its internal consistency was 0.92 in sample 1 and 0.90 in sample 2.

The *Index of General Job Satisfaction* (*GJS;* Fischer and Lück, 1972) is a questionnaire in German language and measures job satisfaction very broadly. It consists of two items, which do not relate to specific aspects of a job (i.e., “I really enjoy my job”; “What do you think: overall, would you say your job is really interesting and satisfying”). Answers are given on a 5-point Likert-scale (from 1 = *untrue* through 5 = *true*). Inter-item correlation is 0.47 (Fischer and Lück, 1972). This measure was chosen to prevent content overlap and inflated correlations with the JSS; more detailed job satisfaction measures ask for similar topics. In the present study, inter-item correlations were 0.68 and 0.69 in sample 1 (mixed sample) and 2 (nurses sample), respectively. Responses were averaged to compute the scale score for job satisfaction.

### PROCEDURE

Sample 1 (mixed sample) was recruited in several ways to obtain a heterogeneous sample (e.g., flyer distributed in city center, snowball system via email and social networks). Sample 2 (nurses sample) was recruited via information on the Website of the Swiss professional association of nurses and via press coverage in a journal for nursing. The only requirement for participation was to work at least 50% of full time hours. All participants completed the questionnaires and provided information on demographics via the Internet. Respondents were not paid for participation, but were given a feedback of individual results.

## RESULTS

### DESCRIPTIVE STATISTICS FOR THE MEASUREMENTS (VIA-IS FACTORS, SVF-120 STRATEGIES, JSS, AND GJS) IN THE TWO SAMPLES

For an examination of the measurements, mean, standard deviation, skewness, and kurtosis were computed for all scales in each of the two samples. Furthermore, reliability analyses (Cronbach’s alpha) were conducted (see **Table 2**).

**Table 2:** Descriptive statistics, and reliability of the VIA-IS factors, SVF120 strategies, JSS, and GJS in sample 1 (mixed sample) and sample 2 (nurses sample).

	Sample 1 (mixed sample)					Sample 2 (nurses sample)				
	*M*	*SD*	*Sk*	*K*	α	*M*	*SD*	*Sk*	*K*	α
**VIA-IS strengths factors**
Emotional	–0.07	1.04	–0.30	–0.08	—	0.08	0.95	–0.14	0.04	—
Interpersonal	0.04	1.07	–0.33	0.35	—	–0.05	0.91	–0.34	0.55	—
Restraint	–0.03	1.05	–0.21	–0.11	—	0.04	0.94	–0.26	1.17	—
Intellectual	0.03	1.03	–0.08	–0.33	—	–0.04	0.96	0.10	–0.33	—
Theological	–0.07	1.04	0.05	0.04	—	0.08	0.95	0.16	1.15	—
**SVF120 strategies**
POS	12.51	2.23	–0.44	0.08	0.92	12.40	2.11	0.53	0.97	0.91
POS1	10.60	2.91	–0.10	–0.08	0.87	10.00	2.61	0.52	0.85	0.84
POS2	11.41	2.96	–0.21	0.00	0.88	11.79	2.73	0.55	0.84	0.87
POS3	15.88	2.63	–0.38	0.20	0.84	15.60	2.67	0.09	–0.09	0.85
NEG	10.38	3.45	0.38	0.12	0.95	10.46	3.55	0.32	–0.06	0.96
**Stress and job satisfaction**
JSS frequency	4.54	1.30	0.36	0.18	0.92	4.59	1.13	0.11	–0.26	0.90
GJS	3.79	0.95	–0.92	0.87	0.80	4.00	0.87	–0.96	0.77	0.82

*N*_Mixed sample_ = 214 (71 men, 143 women); *N*_Nurses_ = 175 (11 men, 164 women). VIA-IS, Values in Action Inventory of Strengths; SVF120, Stress Coping Inventory; POS, positive coping strategies; POS1, devaluation/defense; POS2, distraction; POS3, control; NEG, negative coping strategies; JSS frequency, frequency subscale of the Job Stress Survey; GJS, Index of General Job Satisfaction. An em dash (–) indicates that the scores were not computed (factor scores).

**Table 2** shows that skewness and kurtosis indicated normal distribution of all scales in both samples. Standard deviation showed the tendency to be smaller in the more homogeneous sample 2 (nurses). Internal consistencies were satisfying. The means of the SVF120 scales and the JSS Frequency subscale ranged around the scale midpoints (i.e., 12 for the SVF120; 4.5 for the JSS). Mean of the GJS was considerable above the scale midpoint of 2.5 in both samples (i.e., minus one standard deviation was still above 2.5).

Several analyses were conducted to examine the differences between the two samples. A MANCOVA was computed with sample (sample 1 vs. sample 2) as between-subject factor, demographics (i.e., gender, education, and percentage of employment) as covariates, and character strengths factors, coping strategies, job satisfaction, and frequency of stress as dependent variables. Results indicated significant differences between the two samples in the dependent variables, *F*(11, 374) = 2.72, *p* = 0.002, partial η^2^ = 0.074. Subsequently conducted ANCOVAs showed that nurses (sample 2) were more satisfied with their jobs than the participants in the mixed sample (sample 1; *F*[1, 384] = 7.15, *p* = 0.008, partial η^2^ = 0.018; *M*_Sample 1_ = 3.75 vs. *M*_Sample 2_ = 4.04 corrected for covariates). Furthermore, nurses (sample 2) reported higher frequency of stress than the mixed sample of employees (sample 1; *F*[1, 384] = 7.85, *p* = 0.005, partial η^2^ = 0.020; *M*_Sample1_ = 4.46 vs. *M*_Sample2_ = 4.69 corrected for covariates). Overall, there were a few, but meaningful differences with small effect sizes (cf. Cohen, 1988) between the two samples.

### RELATIONSHIPS BETWEEN CHARACTER STRENGTHS FACTORS AND COPING STRATEGIES

For an examination of the relationships between character strengths and coping, partial correlations (controlled for gender, education, and percentage of employment) between the SVF120 (coping strategies) and the VIA-IS (character strengths factor scores) were computed. **Table 3** shows the correlation coefficients for each of the two samples.

**Table 3:** Partial correlations (controlled for gender, education, and percentage of employment) between character strengths (VIA-IS factors) and coping (SVF120 strategies).

SVF120	Emotional	Interpersonal	Restraint	Intellectual	Theological
**Sample 1 (mixed sample)**
Positive coping strategies (POS)	0.14*	0.11	–0.02	0.38***	0.16*
Devaluation/defense (POS1)	0.14*	0.08	–0.07	0.28***	0.00
Distraction (POS2)	0.00	0.02	–0.01	0.28***	0.22**
Control (POS3)	0.26***	0.20**	0.03	0.36***	0.12
Negative coping strategies (NEG)	–0.03	–0.25***	0.03	–0.19**	–0.01
**Sample 2 (nurses sample)**
Positive coping strategies (POS)	0.22**	0.36***	0.09	0.40***	0.16*
Devaluation/defense (POS1)	0.12	0.19*	0.09	0.39***	–0.01
Distraction (POS2)	0.07	0.32***	0.06	0.29***	0.23**
Control (POS3)	0.36***	0.32***	0.08	0.30***	0.11
Negative coping strategies (NEG)	–0.13	–0.26***	0.16*	–0.16*	–0.09

*N*_Mixed sample_ = 214 (71 men, 143 women); *N*_Nurses_ = 175 (11 men, 164 women). VIA-IS, Values in Action Inventory of Strengths; SVF120, Stress Coping Inventory. *p < 0.05; **p < 0.01; ***p < 0.001.

**Table 3** shows that the relationships between coping and character strengths factors were similar across the two samples. As expected intellectual, emotional, and interpersonal strengths were positively related with positive coping strategies (POS); however, in sample 1 the correlation between interpersonal strengths and positive coping was not statistically significant although being in the right direction. Intellectual and interpersonal strengths were negatively related to negative coping strategies (NEG) as expected, but emotional strengths did not.

As stated in Hypothesis 1, intellectual strengths were positively correlated to the three different subcategories of positive coping. Emotional strengths were statistically more strongly related to control (POS3) than to devaluation/defense (POS1) and distraction (POS2) in both samples as expected in Hypothesis 2 (all *p* < 0.001 one-tailed; except difference between the correlation coefficients of POS3 and POS1 where *p* < 0.05 one-tailed, cf. Steiger, 1980). Interpersonal strengths were related to all coping strategies (i.e., POS, POS1, POS2, POS3, NEG) in sample 2 (nurses), but this was not the case in sample 1 (mixed sample). In sample 1 POS3 and NEG were significantly associated with interpersonal strengths. In line with Hypothesis 3, interpersonal strengths were more strongly related to the positive coping strategies (POS) in sample 2 (nurses sample) than in sample 1 (mixed sample); the difference between the two correlation coefficients was significant (*p* < 0.01, one-tailed). This could be traced back to the numerically (but not statistically significantly) higher correlation coefficients for devaluation/defense (POS1; difference of coefficients: *p* = 0.14, one-tailed) and control (POS3; difference of coefficients: *p* = 0.11, one-tailed) as well as the statistically significantly higher correlation coefficient for distraction (POS2; *p* < 0.01, one-tailed) in sample 2 (nurses sample) compared to sample 1 (mixed sample). Strengths of restraint seemed to be of low relevance for the coping strategies. Theological strengths tended to be related to the positive coping strategy of distraction (POS2) in both samples, which also might have led to the positive correlation with positive coping strategies (POS).

### WORK-RELATED STRESS, CHARACTER STRENGTHS, AND JOB SATISFACTION

For an examination of the relationships among frequency of work-related stress, character strengths, and job satisfaction, several steps of analyses were undertaken. Firstly, participants of the total sample were grouped into three stress-level groups (1 = low level, 2 = medium level, 3 = high level) using the anchors of the rating scale as cut-offs (i.e., low = scores lower than 4; medium = scores between 4 and 6; high = scores higher than 6). Secondly, six univariate ANCOVAs for the total sample were computed with stress-level groups as grouping variable, demographics as covariates (i.e., gender, education, percentage of employment), and with job satisfaction and the character strengths factors as dependent variables. Although nurses reported higher frequencies of work-related stress than the mixed sample, analyses yielded no statistically significant interaction effects between stress-group and sample on job satisfaction and the strengths factors. Hence, analyses were computed utilizing the whole sample with a higher statistical power (sample sizes of three stress-level groups were *n*_low_ _stress_ = 126, *n*_medium_ _stress_ = 214, and *n*_high_ _stress_ = 49), but also for each sample separately in order to provide a more comprehensive presentation of the results. Furthermore, results of ANCOVAs did not change, when covariates were not considered.

The stress level-groups showed significant differences in the intellectual strengths (*F*[2, 383] = 6.08, *p* < 0.01, partial η^2^ = 0.03) and in job satisfaction (*F*[2, 383] = 9.00, *p* < 0.001, partial η^2^ = 0.05). Intellectual strengths increased with the frequency of work-related stress (*M*_low stress group_ = –0.17 vs. *M*_medium stress group_ = 0.01 vs. *M*_high stress group_ = 0.39; low stress group and high stress group differed significantly from each other). Job satisfaction decreased with the frequency of work-related stress (*M*_low stress group_ = 4.12 vs. *M*_medium stress group_ = 3.82 vs. *M*_high stress group_ = 3.51; all groups differed significantly from each other). Results did not differ when splitting the total sample into three groups with equal sizes using the scores on the percentiles 33 and 66% what might be interpreted as a sign of the robustness of the results. Furthermore, results were highly similar when analyzing each of samples separately. Nevertheless, due to smaller sample sizes and consequently lower statistical power, some of the main effects were only marginally significant when analyzing each of samples separately (i.e., *p*-values for differences in the intellectual strengths were *p* = 0.052 and 0.086 in the mixed sample and in the nurses sample, respectively).

#### Character strengths as mediators in the relationship between stress and job satisfaction

**FIGURE 1:**
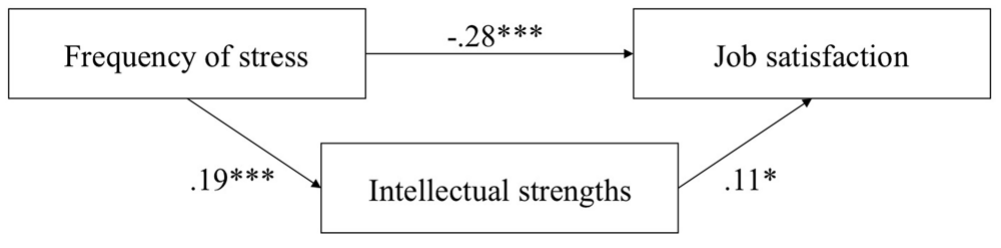
Regression model of the effect of frequency of stress on job satisfaction, which is partially mediated by intellectual strengths; *F* (5, 383) = 6.64, *p* < 0.001. *p < 0.05; ***p < 0.001.

As only the intellectual strengths were related to the frequency of work-related stress, only this character strengths factor met the requirement for a mediation analysis defined by Baron and Kenny (1986). Therefore, the examination of the mediation effect was conducted for the intellectual strengths, but not for the remaining character strengths factors. To examine whether intellectual strengths mediated the link between frequency of work-related stress and job satisfaction a path analysis was computed (utilizing Preacher and Hayes’, 2008, indirect procedure) utilizing the total sample. The independent variable was frequency of work-related stress, mediator was the factor intellectual strengths, and the dependent variable was job satisfaction. Again, gender, education, and percentage of employment were the covariates. The results for the interplay between frequency of work-related stress, intellectual strengths, and job satisfaction are shown in **Figure 1**.

**Figure 1** shows that high frequency of work-related stress was related to low scores in job satisfaction. Furthermore, this association was mediated by intellectual strengths as expected in Hypothesis 4. Intellectual strengths increased with the frequency of stress. Furthermore, job satisfaction was higher with enhanced intellectual strengths. The mediation was a partial (and not a full) one, because there was still a significant association between frequency of stress and job satisfaction. Results were highly similar when analyzing each of samples separately. Nevertheless, due to smaller sample sizes and consequently lower statistical power, the path from intellectual strengths to job satisfaction did not reach statistical significance when analyzing each of the samples separately.

#### Character strengths as moderators in the relationship between stress and job satisfaction

Five hierarchical multiple regressions were computed to test the moderating effect of character strengths factors (i.e., one regression analysis for each of the factors). The control variables (i.e., gender, education, percentage of employment) were entered first, the main effect variables (i.e., z-scores of frequency of work-related stress and the character strengths factor of interest) entered in a second step, and the interaction term between z-scores of frequency of work-related stress and the character strengths factor in a third step. The interaction term must be significant in order to support the moderator hypothesis (Baron and Kenny, 1986). Hierarchical multiple regressions did not yield any significant interaction term (neither in the two samples individually nor in the whole sample). Therefore, character strengths were not moderators here and Hypothesis 5 could not be confirmed.

## DISCUSSION

The present study was aimed at examining the role of character strengths as positive personality traits in dealing with stress. One of the core characteristics of character strengths is, that they determine “how an individual copes with adversity” (Peterson and Seligman, 2004, p. 17). Therefore, systematic relationships between character strengths and coping behavior were expected. Data presented from two samples (i.e., a sample of employees from various occupations and a sample of nurses) showed that character strengths (1) were systematically related to coping, and (2) mediated the effects of work-related stress on job satisfaction. Intellectual strengths were especially related to coping followed by emotional, and interpersonal strengths. Strengths of restraint and theological strengths were of little relevance for dispositional coping behavior. This is in line with the expectations derived from previous publications on coping with adversity (i.e., Peterson and Seligman, 2003; Peterson et al., 2006, 2008) and from content-driven assumptions.

Intellectual strengths were the ones most strongly associated with coping with work-related stress. They correlated with positive coping and every subcategory of it (i.e., devaluation/defense, distraction, control) as well as with negative coping in the intended direction. These results highlighted the importance of intellectual strengths for dispositional coping behaviors. Love of learning, judgment, curiosity, and creativity are components of the intellectual strengths factor (cf. Ruch et al., 2010). All those character strengths foster the production of new and reasonable strategies for problem solving and the exploration of situational circumstances (cf. Peterson and Seligman, 2004), what in turn assists in the selection of the most successful coping strategies (i.e., positive coping) and the avoidance of unsuccessful coping strategies (negative coping). That might also explain, why intellectual strengths mediated the negative effect of work-related stress on job satisfaction. Additionally, challenges experienced during stress might foster intellectual strengths, which in turn are positively associated with job satisfaction. However, due to the cross-sectional design of the present study, conclusions regarding causality could not be drawn, and another causal direction could be assumed as well. For example, people with higher intellectual strengths might have a better education (cf. Ruch et al., 2010) and therefore work in jobs with more responsibility, which is associated with more stress. However, education served as a control variable in all analyses and results still emerged. Nevertheless, studies utilizing longitudinal and intervention designs are needed to address research questions regarding causality.

Interpersonal strengths were negatively related to negative coping (NEG) on both samples. People who see the bright side of life (humor) and have good relationships (because of their kindness) might show a lesser tendency to escape, withdraw from social contacts, ruminate, and give up. Furthermore, interpersonal strengths showed different correlation pattern with respect to positive coping strategies in a sample of nurses and a mixed sample (participants with different occupations). Especially the positive coping strategy distraction (POS3) was stronger related to interpersonal strengths in nurses than in the mixed sample. It has been highlighted that the (sometimes) problematic contact with doctors as well as patients and their relatives is an often-observed, characteristic stressor for nurses (e.g., Harris, 1989; Burgess et al., 2010). Hence, seeking distraction from this kind of strains might be very likely among nurses. Distraction can be achieved, for example, by focusing on someone that who is creating a situation incompatible with stress (Janke et al., 1985; Janke and Erdmann, 2008). This behavior might profit from interpersonal strengths that might help to create a kind and humorous atmosphere in situations with colleagues and friends what in turn helps to relax and distance from situations characterized by problematic conflict with others like the patients and their relatives.

Emotional strengths were found to be related to positive coping but less so to negative coping. Emotional strengths include active behaviors (e.g., being brave, persistent, and hopeful, having perspective), which foster an effective analysis of the situation and problem solving (i.e., positive coping strategies). This is in line with results reported by Gander et al. (2012), who found that an active, offensively minded work-related attitude toward obstacles and challenges was strongly associated with emotional strengths. Emotional strengths seem to assist controlling one’s own reactions, and facing a stressful event directly rather than engaging in a more passive distraction or withdrawal, escape, rumination, and self-blame.

Theological strengths were related to distraction coping (POS2) in both samples. These character strengths include behaviors like being grateful, seeing the beauty, and meditation (Peterson and Seligman, 2004). Focusing on what one is thankful for and meditation should foster relaxation and distraction. Furthermore, appreciation of beauty and excellence is related to the disposition to experience positive emotions like joy and awe (cf. Güsewell and Ruch, 2012), what might also foster distraction coping (POS2).

### LIMITATIONS AND FUTURE RESEARCH

This study has several limitations that should be mentioned. First, because the data are self-reported, common method variance may have inflated correlations (cf. Doty and Glick, 1998). However, correlation pattern between coping and character strengths varied across the coping scales and the character strengths factors. Therefore, it was concluded that the results were not overly affected by this bias. Furthermore, self-ratings were the chosen source of data in the present study, because co-workers might not be able to provide a full reflection of the self-raters’ possession of the character strengths. As people might just show certain strengths at work due to the formal requirements and restrictions (cf. Ten Berge and De Raad, 1999; Harzer and Ruch, 2013), it would be difficult to ascertain that everyone has a coworker that knows him or her well enough. Therefore, the validity of a peer-rating might be challenged. Furthermore, as the experience of stress frequency and the use of coping strategies were considered to be in large parts intra-individual experiences, the self-ratings were considered the most valid judgments. Nevertheless, future studies could utilize multiple data sources to eliminate the effects associated with common method variance.

Second, aiming at investigating the relationships between character strengths and coping in general, a cross-sectional design was chosen. However, the cross-sectional design did not allow any conclusions about causal relationships between the variables. Although causal directions and mechanisms were formulated in the paper occasionally in order to describe the assumed role of character strengths for coping with stress, studies utilizing longitudinal and intervention designs are needed to address research questions regarding causality. The cross-sectional design in combination with the low rate of unsatisfied participants in the presented data might have caused that there was no moderation effect for character strengths on the relationship between work-related stress and job satisfaction. Additionally, utilizing the two-item job satisfaction scale instead of a more extensive one used in the present study might have prevented the detection of interaction effects. Frameworks for studying personality in the stress process assume a moderating role of personality traits on the relations between stressor and outcomes like job satisfaction (cf. Bolger and Zuckerman, 1995). Personality influences the reactivity (i.e., emotional and physical reactions) within a stressful event. However, most studies examining the role of certain personality traits are diary studies (e.g., Bolger and Zuckerman, 1995; Hahn, 2000). The present study presented cross-sectional, self-rating data from samples moderate in size; the data therefore did not seem to be able to illustrate this process. Further research might study the process of coping with a stressful event and the role of character strengths within this process. For a further examination of the role of character strengths within the stress (and coping) process, the framework by Bolger and Zuckerman (1995) provides promising ideas. For example, it can be expected that character strengths influence the exposure to certain stressors, and that interpersonal strengths may lead to more social contacts. Moreover, intellectual strengths may help to judge a stressful event more rationally and hence, lower the negative effect of stress on outcomes like job satisfaction. Additionally, character strengths may influence the decision of whether or not to use a specific coping strategy, and therefore the effectiveness of coping in a specific situation.

Third, the aim of the present study was to get a general overview on the relationships between character strengths and coping. Therefore, analyses were conducted on a very broad level of five character strengths factors and four coping scales. Given the fact, that on the most narrow level 24 character strengths and 20 coping modes are assessed in the measures utilized here, much more fine-grained investigations could be conducted in the future with multiple-source data from larger samples.

## CONCLUSION

One approach to reduce the impact of work-related stress is to decrease the frequency of stressors. However, this might not be always possible. In the light of the present study, character strengths as trainable personal characteristics (Peterson and Seligman, 2004) seem to be important resources for the training on and/or off the job to improve coping with work-related stress. Studies have shown that character strengths can be fostered by systematic interventions (e.g., Gander et al., 2013; Proyer et al., 2013b). Fostering character strengths in employees might lead to a decrease in the negative consequences of work-related stress, because employees might be better able to cope with it. That might have a positive impact on the employees’ job satisfaction, but also productivity, lowered absenteeism, and job performance (e.g., Landsbergis, 1988; Karasek and Theorell, 1990; Cooper and Cartwright, 1994; Harzer and Ruch, 2014). Furthermore, the results presented in the paper at hand might also be interpreted with respect to the implications for personnel selection. For example, when assigning (new) employees to positions with higher stress frequency, recruitment procedures might be designed to consider the level of character strengths especially relevant for coping with stress as well in order to lower the chance of negative consequences of work-related stress.

Overall, the present study underlined that character strengths relate to how individuals deal with adversities (in the workplace). They are associated with the strategies utilized by individuals to cope with stress, and buffer the negative effects of work-stress on job satisfaction. These findings open a new field for research on the role of personality (here: character strengths as positive traits) in coping with work-related stress. Further research is needed on the role of character strengths within the process of coping with a stressful event.

## Conflict of Interest Statement

The authors declare that the research was conducted in the absence of any commercial or financial relationships that could be construed as a potential conflict of interest.
